# A case study of the use of deception in brief intervention research: an ethical evaluation

**DOI:** 10.1186/1940-0640-8-S1-A47

**Published:** 2013-09-04

**Authors:** Jim McCambridge

**Affiliations:** 1 Faculty of Public Health, London School of Hygiene & Tropical Medicine, UK

## Background

Some brief intervention trials involve deception through blinding. A methodological imperative to minimise bias can be in conflict with the ethical principle of informed consent. This presentation describes efforts over a period of some years to address a set of longstanding methodological and ethical issues in the field of brief alcohol intervention trials.

## Objective

To undertake an ethical evaluation of research practice.

## Methods

The specific forms of deception used in three online trials of brief alcohol interventions are examined in a case study. These studies have common features, involving thousands of university students receiving an e-mail with study participation being triggered by responding to this e-mail.

## Results

This case study is located within the wider literature on the use of deception in research and within the context of evolving approaches to public health ethics. Decision making about the use of deception, is presented along with ethical justifications and ongoing uncertainties. The value of the approach of *pragmatism* for examining these kinds of ethical issues is considered.

## Conclusions

The use of deception in brief intervention research and elsewhere should be treated with scepticism on ethical grounds. It should not, however, be rejected out of hand. Its possible use should be considered carefully by ethical committees, paying close attention to study context. If it is judged useful or necessary to produce more valid inferences, the moral costs involved in obtaining such data need to be considered in relation to the moral benefits that such data may produce, which are in turn contingent upon the scientific and social value of the research. Evaluation of the costs and benefits will be enhanced by empirical data.

